# TD Swin-UNet: Texture-Driven Swin-UNet with Enhanced Boundary-Wise Perception for Retinal Vessel Segmentation

**DOI:** 10.3390/bioengineering11050488

**Published:** 2024-05-14

**Authors:** Angran Li, Mingzhu Sun, Zengshuo Wang

**Affiliations:** College of Artificial Intelligence, Nankai University, Tianjin 300350, China; sunmz@nankai.edu.cn (M.S.); 2120210391@mail.nankai.edu.cn (Z.W.)

**Keywords:** retinal vessel segmentation, transformer, cross-level texture complementary module, pixel-wise texture highlighting module, Hausdorff loss

## Abstract

Retinal vessel segmentation plays a crucial role in medical image analysis, aiding ophthalmologists in disease diagnosis, monitoring, and treatment guidance. However, due to the complex boundary structure and rich texture features in retinal blood vessel images, existing methods have challenges in the accurate segmentation of blood vessel boundaries. In this study, we propose the texture-driven Swin-UNet with enhanced boundary-wise perception. Firstly, we designed a Cross-level Texture Complementary Module (CTCM) to fuse feature maps at different scales during the encoding stage, thereby recovering detailed features lost in the downsampling process. Additionally, we introduced a Pixel-wise Texture Swin Block (PT Swin Block) to improve the model’s ability to localize vessel boundary and contour information. Finally, we introduced an improved Hausdorff distance loss function to further enhance the accuracy of vessel boundary segmentation. The proposed method was evaluated on the DRIVE and CHASEDB1 datasets, and the experimental results demonstrate that our model obtained superior performance in terms of Accuracy (ACC), Sensitivity (SE), Specificity (SP), and F1 score (F1), and the accuracy of vessel boundary segmentation was significantly improved.

## 1. Introduction

Medical image segmentation plays a pivotal role in medical image processing and analysis, encompassing tasks such as brain segmentation, cell segmentation, lung segmentation, retinal blood vessel segmentation, and so on. Precise segmentation can facilitate accurate lesion localization, thus aiding doctors in formulating optimal treatment plans. With the continuous advancements in computer vision and artificial intelligence, medical image segmentation finds extensive application in ophthalmology, among which the retinal vascular segmentation field has attracted much attention. Retinal vessel segmentation technology enables the analysis of vascular structures in fundus images, providing an important reference for early diagnosis and treatment of ophthalmic diseases.

Retinal vascular segmentation is of great importance to the diagnosis of ocular diseases. The observation of abnormal structures in retinal vascular segmentation images can help physicians detect signs of common eye diseases such as diabetic retinopathy, glaucoma, and macular degeneration in a timely manner [1], leading to more timely interventions and therapeutic measures. Nonetheless, retinal vessel segmentation and visualization present challenges due to vessel size variability, intertwined branches, and complex structures [2]. Consequently, there is a pressing need for automated and efficient retinal vessel segmentation methods to enhance ocular disease diagnosis and treatment while enabling the automated analysis of medical images.

Traditional retinal vessel segmentation methods typically include threshold segmentation algorithms, matched filtering algorithms, and machine learning-based approaches. The threshold segmentation method [3] is straightforward but sensitive to factors like image noise and illumination changes, requiring the re-selection of thresholds for different images and applications. Matched filtering algorithms [4] leverage 2D convolution operations with filters constructed based on 2D Gaussian templates to capture blood vessel features. Matched filtering algorithms are robust to image noise and illumination variations, but they require manually designed filters and parameters and struggle to handle complex situations such as blood vessel crossings and overlaps. Machine learning-based methods utilize manually crafted features (edges, texture, color, etc.) to discern vascular from non-vascular regions, which are subsequently segmented by a classifier. Despite their potential, this approach heavily relies on feature selection and extraction, demanding considerable human intervention and empirical knowledge, thus limiting adaptability across various retinal image types.

Deep learning has witnessed a surge in popularity owing to enhanced computational capabilities, the availability of large-scale datasets, and algorithmic advancements. Convolutional neural networks (CNNs) have achieved remarkable achievements across various fields, including image classification [5], target detection [6], and image segmentation [7]. In image segmentation, CNNs have significant advantages over traditional methods by automatically extracting features and learning complex representations with robust adaptability and generalization. Several image segmentation networks have been proposed, including SegNet [8], UNet [9], PSPNet [10], and UNet++ [11]. UNet introduces an encoder–decoder structure along with a skip-connection mechanism. UNet++ enhances UNet by incorporating a dense connection mechanism and constructing a multi-layered skip connection. The UNet or improved UNet network integrates low-resolution information such as object categories and high-resolution information such as edges and details, making it suitable for medical image segmentation tasks. U-Net and its variants have been widely used in retinal blood vessel segmentation tasks [12,13,14].

The above CNN models have achieved remarkable results, but the convolution operation can only capture local spatial information, while lacking robustness in capturing global contextual information. In fundus vessel segmentation tasks, distant pixels may exhibit correlations with local image structures. CNNs struggle to model global information and long-distance interaction information, thus ignoring the continuity and wholeness between blood vessels, and hence still represent a major limitation in retinal vessel segmentation. In order to realize global contextual information capture, the transformer [15] framework was then proposed. Utilizing a self-attentive mechanism [16], transformers can capture the correlation among all positions in a sequence at each time step and use it to compute the output, which can effectively deal with long-distance dependencies in a sequence. Initially applied in natural language processing, transformers found success in image classification with the proposal of the Vision Transformer (ViT) [17]. However, due to extensive computational costs, the substantial parameter count and high computational demands of the ViT present great challenges. Swin Transformer [18] introduced the windowed self-attention mechanism, partitioning the input image into fixed-size blocks for self-attention computation, thereby reducing computational complexity. Inspired by the U-shaped encoder–decoder structure of U-Net, Swin-UNet [19] was developed and achieved notable success in medical image segmentation.

Swin-UNet, as a semantic segmentation network based on the Swin Transformer, performs well in processing large-scale images and regular data. However, it encounters challenges when segmenting retinal blood vessel images, characterized by numerous small regions and dense boundaries. We summarize the problems of the Swin-UNet retinal blood vessel segmentation method as follows: (1) During gradual downsampling and upsampling, as the size of the feature map decreases, the network forfeits the shallow details containing richer semantic features of small regions, particularly texture features at the boundaries of the target region. These details are crucial for segmentation tasks, aiding in distinguishing between the intersections of multiple region categories and enhancing segmentation quality. Once this information is lost, the network may not be able to fully recover the lost details of boundaries and contours in the upsampling stage despite integrating shallow features with the deeper ones through skip connections, leading to the decrease in the segmentation ability. (2) The Swin-UNet loss function employs cross-entropy and Dice losses. While Dice loss suits unbalanced category segmentation tasks, it proves highly unstable for regions with small areas, notably the minuscule regions in retinal blood vessel images, thereby diminishing model segmentation performance to a certain extent. Dice loss solely quantifies discrepancies between predictions and ground truth in terms of pixel count, disregarding specific boundary shapes and distributions. There are only foreground and background parts in the retinal blood vessel image, and the latter are very small. Inaccuracies in predicting some pixels in these regions may trigger significant changes in Dice loss, resulting in drastic gradient shifts and ultimately affecting model performance.

To address the above problems, we improve Swin-UNet and introduce TD Swin-UNet. Specifically, to tackle the limited capability in localizing and segmenting the boundaries of Swin-UNet, we propose the texture-driven retinal vessel segmentation method by improving boundary-wise perception. Previous studies have demonstrated that shallow semantic features contain richer textures, such as boundaries and contours, due to their higher resolution, which substantially aids in model performance. Consequently, we concatenate the outputs of multiple Swin Blocks in the network encoder and feed them into a Cross-level Texture Complementary Module (CTCM). This module amalgamates these feature maps to further augment the model’s ability to extract and represent semantic features such as boundaries and contours. Moreover, we enhance the Swin Block in the decoder and introduce the Pixel-wise Texture Swin Block. This module heightens the model’s focus on the vicinity of the region boundary, thereby improving boundary localization and segmentation performance. To address the insensitivity of the Dice loss function to the specific shape and distribution of boundaries, we introduce the Hausdorff distance loss and refine the Hausdorff loss by incorporating a clip truncation operation to avoid the imbalance due to the size of the area, ultimately enhancing the model accuracy in segmenting blood vessel boundaries. Our proposed model demonstrates outstanding segmentation results on two datasets: DRIVE and CHASEDB1. The primary contributions of this paper are summarized as follows:

(1) We propose a texture-driven retinal vessel segmentation method by improving boundary-wise perception, which contains two key enhancements. Firstly, we introduce the Cross-level Texture Complementary Module (CTCM) to fuse feature maps during the encoding process, facilitating the focus of our model on essential feature information in vessel images and recovering the shallow details lost during the downsampling stages. Additionally, we introduce the Pixel-wise Texture Swin Block (PT Swin Block) via the Pixel-wise Texture Highlighting Module (PTHM), which enhances the model’s capacity to perceive and recognize vessel boundary and contour information;

(2) We improve the loss function by introducing a proposed Hausdorff distance loss function tailored for small target regions of blood vessels. Furthermore, we refine the Hausdorff loss by introducing hyperparameters to weight different components of the loss function. This enhancement enables the model to better discern subtle features of blood vessel structure and boundary information;

(3) We conducted experiments on two datasets: DRIVE and CHASEDB1. The experimental results show that our proposed network outperforms the existing methods on the retinal blood vessel segmentation task.

## 2. Related Works

Medical imaging is an important and indispensable tool in medical diagnosis and treatment. In the past, medical images were primarily analyzed manually by physicians or using simple feature extraction algorithms, which were time-consuming and prone to low accuracy rates. However, in the 21st century, the intelligent analysis of medical image has emerged as a prominent research field. Deep learning technology offers numerous applications in this field, including image classification (X-rays, CT scans, MRIs, and so on), lesion detection (tumors, nodules, blood vessels, and so on), and precise tissue structure or lesion segmentation from the background. The integration of deep learning into medical imaging holds significant promise, as it can provide more accurate, efficient, and intelligent solutions for medical image analysis, ultimately enhancing the accuracy of medical diagnoses and the effectiveness of treatment.

### 2.1. Retinal Vessel Segmentation Networks Based on CNN

In recent years, numerous CNN-based approaches have emerged for retinal blood vessel segmentation, primarily relying on U-Net and its variants as the foundational architecture. U-Net has gained popularity due to its efficacy in capturing spatial information and adept handling of semantic segmentation tasks. Lv et al. [20] proposed AA-UNet, incorporating an attention mechanism to automatically prioritize regions pertinent to blood vessels. Atrous convolution was employed to expand the model’s receptive field, enhancing the capture of vessel details and contextual information. Yang et al. [21] proposed MR-UNet, featuring a multi-scale convolution (Multiconv) block formed by different-sized convolution kernels. They replaced the 3 × 3 convolution block with a Multiconv block to facilitate feature extraction across varying vessel thicknesses and finenesses, and also added residual convolution to the skip connection, reducing the information difference between the encoder and decoder. Radha et al. [22] augmented the encoding process with a deepwise convolution block to mitigate information decay during downsampling and introduced a parallel attention network in the upsampling process to optimize the model structure. Cao et al. [23] optimized the skip connection mechanism of U-Net, incorporating a Multi-scale Fusion Self-Attention Module to leverage diverse image scales. They also replaced the original output layer with a Multi-branch Decoding Module (MBDM) to construct MFA-UNet, enabling more detailed vessel segmentation.The above models have achieved remarkable results, but CNNs can only capture local spatial information and are unable to comprehend global information. To address this limitation, we leverage transformer as the framework for segmentation networks.

### 2.2. Retinal Vessel Segmentation Networks Based on Transformer

Compared to CNN models, transformers exhibit a stronger ability to capture global positional information, which enables better attention to the entirety and continuity of blood vessels, resulting in improved segmentation effectiveness. Jiang et al. [24] enhanced the Multi-head Self-Attention (MSA) mechanism in a transformer and introduced the Transformer Positional Attention (TPA) module to precisely acquire the position information of blood vessel pixels. They integrated TPA with UNet during the encoding process and proposed the MTPA-UNet model. Jiang et al. [25] fused a CNN with the ViT and built CoVi-Net. They designed the LGFA architecture to capture long-range feature dependencies and proposed bidirectional weighted feature fusion (BWF) with adaptive lateral feature fusion (ALFF) for features of varying scales. Jia et al. [26] proposed DT-Net, which merges deformable convolution with the transformer and incorporates MSA in the decoder to capture long-range dependencies and important features within blood vessels. In addition to fusing the transformer with the CNN, some scholars have also focused on enhancing transformer structures or attention mechanisms to improve segmentation accuracy. Tan et al. [27] enhanced the MSA module in the ViT and proposed Multi-Head Dynamic Token Aggregation Attention (MDTAA) to capture the global information of the retina. They also added an auxiliary convolution branch to accelerate the model convergence, ultimately constructing the OCT2Former network for OCTA retinal vessel segmentation. Wang et al. [28] devised an unsupervised blood vessel segmentation method, which resolves optimal mappings using two extreme mapping functions to delineate vascular structures. They employed Swin-UNet to solve the optimal mappings and designed an outlier-aware game filter to mitigate prediction mask errors. Lin et al. [29] proposed SGAT-Net for retinal vascular segmentation, which introduced a Stimulus-Guided Adaptive Module (SGA-Module) to acquire global features. They designed a Stimulus-Guided Adaptive Feature Fusion (SGAFF) module to adaptively emphasize local details and global information, achieving high segmentation accuracy. Transformer-based models are able to capture long-distance dependencies in sequence data and have achieved superior segmentation results. However, when dealing with local pixel-level edges and texture features, these models may overlook the spatial relationships between pixels and fail to accurately capture and segment fine structures near the boundaries, resulting in a decrease in the segmentation and localization accuracy of the blood vessel boundaries, which, in turn, affects the segmentation effect.

## 3. Methods

### 3.1. Overall Framework of the Proposed Network

**Figure 1:**
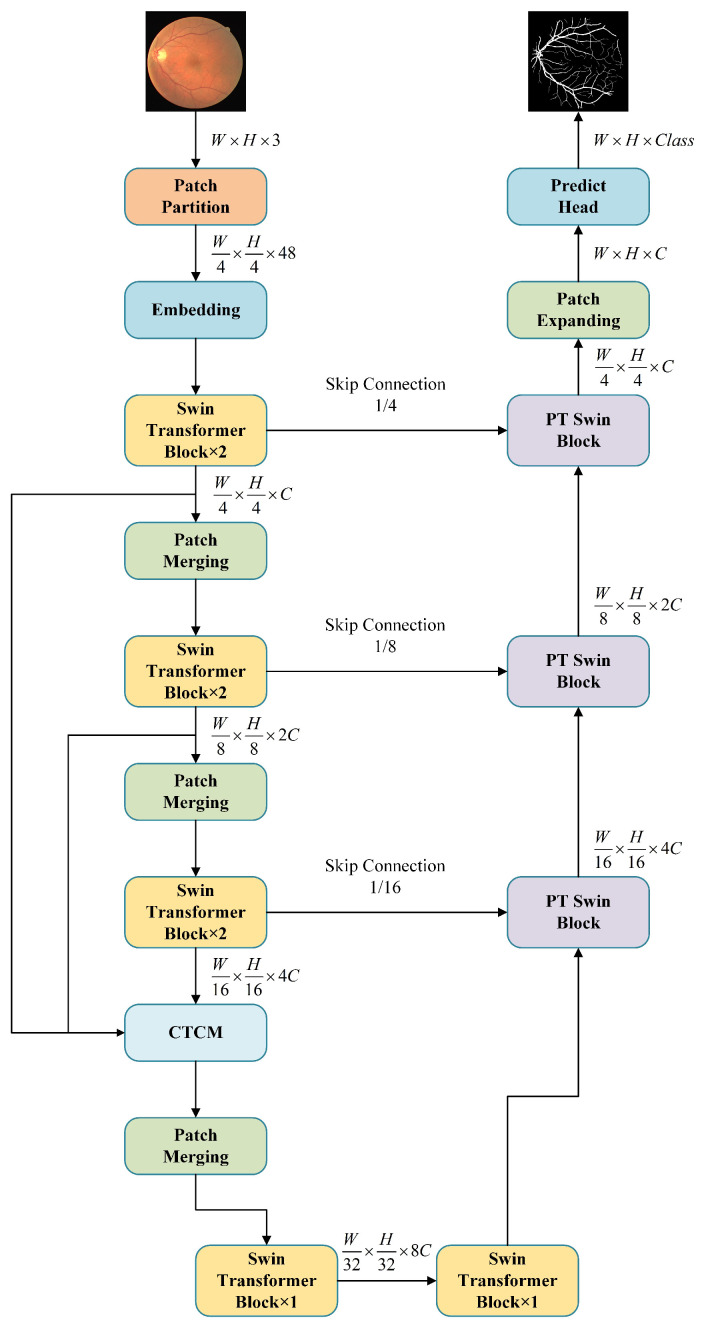
Overall architecture of TD Swin-UNet network.

To address the challenges of key information loss and inaccurate vessel boundary segmentation in Swin-UNet’s downsampling process, we propose TD Swin-UNet. The architecture of TD Swin-UNet is illustrated in Figure 1. TD Swin-UNet is comprised of three key components: the encoder module, the decoder module, and the CTCM module.

In the encoder module, the input image is initially divided into equal-sized image blocks using the Patch Partition module. This operation transforms the *W* × *H* × 3 feature map into $\frac{W}{4}\times \frac{H}{4}\times 48$ vectors. Subsequently, these 48-dimension vectors are projected to *C*-dimension using Linear Embedding and then inputted into the Swin Block. The Swin Block, as the core component of the Swin Transformer, incorporates the shifted window mechanism and allows each position to focus on local neighborhood information, thereby enhancing the capture of spatial local relationships in the image. The output feature map of the Swin Block is connected to the decoder via a skip connection mechanism. Simultaneously, it undergoes downsampling through the Patch Merging module, halving the image height and width while doubling the number of channels. The CTCM module fuses feature maps of different scales in the encoder, enhancing lost boundary contour information and improving the model’s accuracy in boundary segmentation. In the decoder module, we introduce a new PT Swin Block. Based upon the original Swin Block and patch expanding module, the feature maps are enriched with boundary and contour information through the PTHM module, thereby enhancing the model’s ability to learn semantic information on both sides of the boundary. Finally, the segmentation result is obtained through a linear layer.

### 3.2. Swin Transformer Block

**Figure 2:**
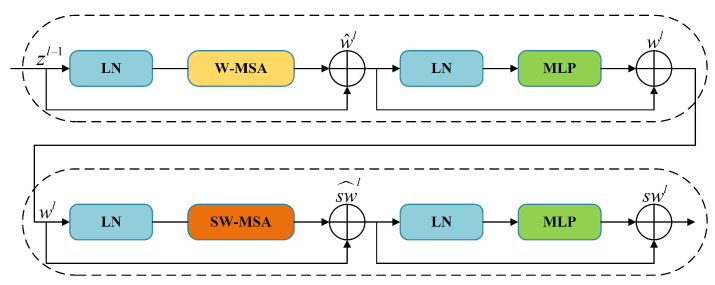
Pipeline of Swin Transformer block.

The Swin Block serves as the basic component in the Swin Transformer, which improves the Multi-Head Self-Attention (MSA) mechanism used in the ViT. It achieves this improvement by employing a windowed attention mechanism and cross-layer local connections to reduce the number of parameters and computational complexity. Specifically, it introduces two variants of attention mechanisms: Window-based Multi-Head Self-Attention (W-MSA) and Shifted Window-based Multi-Head Self-Attention (SW-MSA). The basic structure of the Swin Block is depicted in Figure 2. Each Swin Block comprises two Transformer blocks, with each Transformer block consisting of two LayerNorm (LN) layers and an MLP layer. The W-MSA and SW-MSA modules are applied to the front and back Transformer blocks, respectively. The expressions for these modules are shown below:

(1) $${\hat{w}}^{l}=W\text{\_}MSA\left(LN\left(z^{l-1}\right)\right)+z^{l-1}$$

(2) $$w^{l}=MLP\left(LN\left({\hat{w}}^{l}\right)\right)+{\hat{w}}^{l}$$

(3) $${\hat{sw}}^{l}=SW\text{\_}MSA\left(LN\left(w^{l}\right)\right)+w^{l}$$

(4) $${sw}^{l}=MLP\left(LN\left({\hat{sw}}^{l}\right)\right)+{\hat{sw}}^{l}$$

where $z^{l-1}$ denotes the output of the previous layer, ${\hat{w}}^{l}$, ${\hat{sw}}^{l}$ denote the output of the (S)W-MSA module, and $w^{l}$, ${sw}^{l}$ represent the output of the two MLP modules of layer *l*.

### 3.3. Cross-Level Texture Complementary Module

The encoder part of Swin-UNet contains multiple downsampling sessions, yielding feature maps $F_{1}(F_{1}\in R^{\frac{W}{4}\times \frac{H}{4}\times C})$, $F_{2}(F_{2}\in R^{\frac{W}{8}\times \frac{H}{8}\times 2C})$, and $F_{3}(F_{3}\in R^{\frac{W}{16}\times \frac{H}{16}\times 4C})$ at different scales through forward propagation. To augment the boundary localization and segmentation capabilities of our model, we pass these three feature maps to the Cross-level Texture Complementary Module (CTCM), which sequentially upsamples the lost vessel boundary contour information and weights each channel of the feature maps to prioritize critical feature information, thereby enhancing segmentation accuracy.

**Figure 3:**
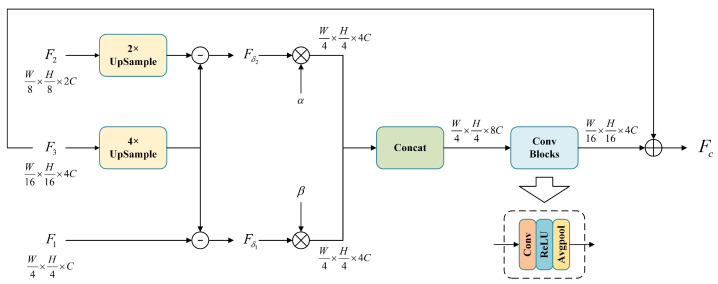
Structure of Cross-level Texture Complementary Module.

The structure of CTCM is shown in Figure 3. Firstly, we upsample $F_{2}$ and $F_{3}$ by two and four times, respectively, to match the height and width of $F_{1}$, and then align the number of channels to 4*C* via a 1 × 1 convolution. However, upsampling alone fails to recover the intricate texture gradually lost during downsampling. Therefore, we introduce two difference values here to recover the detail texture lost in the downsampling process, as shown in Equations (5) and (6).

(5) $$F_{\delta_{1}}=F_{1}-F_{3}^{4\times }$$

(6) $$F_{\delta_{2}}=F_{2}^{2\times }-F_{3}^{4\times }$$

where the superscripts 4×, 2× denote fourfold and twofold upsampling.

To further focus on the importance of $F_{\delta_{1}}$ and $F_{\delta_{2}}$, we introduce two hyperparameters, α and β, to weight the feature maps, respectively, and concatenate two weighted feature maps. Then, the dimensions are reduced to $\frac{W}{16}\times \frac{H}{16}\times 4C$ by the ConvBlocks, which consist of convolution, the ReLU activation function, and average Pooling. During the process, to avoid the recovery of the texture details from being discarded, we chose Average Pooling instead of Max Pooling. Finally, we combine the output with $F_{3}$ to obtain $F_{c}(F_{c}\in R^{\frac{W}{16}\times \frac{H}{16}\times 4C})$. The output of the CTCM module is illustrated in Equation (7).

(7) $$F_{c}=F_{3}+ConvBlocks\left(\left[\alpha F_{\delta_{1}};\;\beta F_{\delta_{2}}\right]\right)$$

### 3.4. Pixel-Wise Texture Swin Block

**Figure 4:**
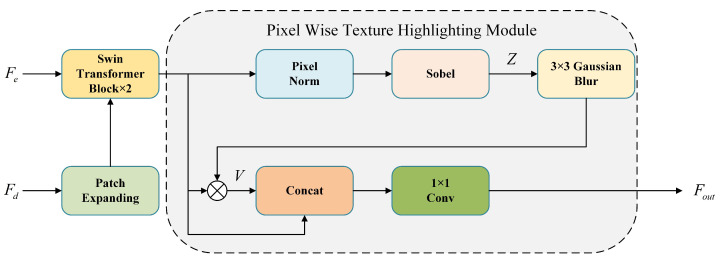
Structure of Cross-level Texture Complementary Module.

We enhanced the decoder structure of Swin-UNet, incorporating three newly designed Pixel-wise Texture Swin Blocks (PT Swin Blocks) as depicted in Figure 4. The Pixel-wise Texture Highlighting Module (PTHM) was developed specifically for retinal vessel segmentation tasks in this study, which aimed to enhance the model’s ability to perceive boundary and contour information of blood vessels, thereby improving the model’s semantic learning on both sides of the boundary and guiding fine-grade segmentation tasks at a higher level. The module takes the feature map $F_{in}\left(F_{in}\in R^{W\times H\times C}\right)$ as input, where *W* and *H* denote the width and height of the feature map, respectively, and *C* represents the number of channels. We first perform Pixel Normalization on $F_{in}$, and then use the Sobel operator to perform convolution in both vertical and horizontal directions to extract the gradient around each pixel location, where larger gradients correspond to boundary regions with richer semantic information. The formulas are shown as below:

(8) $$Z=F_{in}+Sobel\left(PN\left(F_{in}\right)\right)$$

(9) $$Sobel\left(F\right)=\sqrt{{Sobel}_{x}{\left(F\right)}^{2}+{Sobel}_{y}{\left(F\right)}^{2}}$$

where ${Sobel}_{x}\left(F\right)$ and ${Sobel}_{y}\left(F\right)$ denote the convolution results using the horizontal and vertical Sobel operators, respectively. PN denotes pixel normalization, as represented by the following formula:

(10) $$PN\left(F_{x,y}\right)=\frac{F_{x,y}}{\sqrt{\frac{1}{C^{\prime }}\sum_{j=1}^{C^{\prime }}{\left(F_{x,y}^{\left(j\right)}\right)}^{2}+\epsilon }}$$

where $C^{\prime }$ denotes the number of channels of the feature map *F*; $F_{x,y}$ denotes the value of the feature map at position *x*, *y*; and $F_{x,y}^{\left(j\right)}$ denotes the value of the *j*th channel at position *x*, *y*. ϵ is a constant that prevents the denominator from being 0, and has the value of $1\times 10^{-8}$. The purpose of using PN here is twofold. On the one hand, it mitigates the bias introduced by absolute scale differences, ensuring that features from each channel at the same pixel position are thoroughly considered. On the other hand, PN preserves the original semantic relationships between each pixel, maintaining the semantic and textural diversity among different localized regions.

For the feature map *Z*, we aimed for the model to concentrate on the texture of a small localized region near the boundary, because this region is positioned on the contour between categories, assisting the model in accurate localization. At the same time, we also sought to prevent the model from overly focusing on this localized area. Therefore, we applied the Gaussian blur of size 3 × 3 to *Z* and then multiplied it by $F_{in}$ as a weight *G*, as demonstrated in the following formula:

(11) $$V=G⊙F_{in}$$

(12) $$G=GaussianBlur\left(Z,k=3\right)$$

Finally, the final output $F_{out}$ is obtained by concatenating *V* and $F_{in}$, followed by a 1 × 1 convolution to blend the textures across each channel. The formula is shown below:

(13) $$F_{out}=Conv_{1\times 1}\left(\left[V;F_{in}\right]\right)$$

### 3.5. Loss Function Improvement

Apart from the issue of losing boundary texture during the downsampling process in Swin-UNet, its loss function simply uses the summation of cross-entropy loss and Dice loss. The total loss is defined as follows:

(14) $$L_{total}=L_{CE}+L_{Dice}$$

where $L_{CE}$ denotes the cross-entropy loss and $L_{Dice}$ denotes the Dice loss.

To address the insensitivity to the specific shape and boundary distribution of Dice loss, and to further enhance the accuracy of the model predictions for class boundaries in retinal vessel segmentation tasks, it is imperative to introduce a loss term in the loss function that is sensitive to the specific shape of the boundary.

From this perspective, cross-entropy (CE) loss appears to be compliant, as it penalizes the model misclassification of boundary pixels, thereby prompting the model to focus more on the accuracy of segmentation boundaries. Although CE loss can partially consider boundary information, it indirectly influences boundary accuracy through overall pixel classification rather than directly optimizing boundary characteristics. Therefore, its contribution to improving the model’s ability to localize boundary categories is quite limited.

As a result, we introduce the loss term of the Hausdorff distance, denoted as $L_{Haus}$. Suppose that *P* represents the model output and *G* denotes the actual labels. $L_{Haus}$ is defined as below:

(15) $$L_{Haus}=\max\left(Haus\left(P,G\right),Haus\left(G,P\right)\right)$$

where Haus(X,Y) represents the Hausdorff distance between two feature maps *X* and *Y*.

Compared to CE loss, the Hausdorff distance directly measures the disparity between the predicted boundary and the true boundary, offering a more direct metric. The Hausdorff distance aids in capturing subtle differences in boundaries, particularly when pixel-level classification results are ambiguous or when dealing with fuzzy boundaries. It provides a more precise reflection of the distance between the predicted boundary and the true boundary. This is particularly critical in retinal vessel segmentation, where any subtle boundary changes can influence doctors’ assessment of ocular lesions. Therefore, the introduction of the Hausdorff distance enhances the model’s understanding of the specific shape and distribution characteristics of blood vessel boundaries, thereby further improving segmentation accuracy.

However, Haus(X,Y) is significantly influenced by the areas of *X* and *Y*. In the retinal vessel segmentation task, the vessel region and background region areas are often unbalanced. This drawback of Haus(X,Y) tends to result in the loss term of large area targets being too large and the loss term of small area targets being too small, thereby leading to performance degradation. To address this issue, we introduced a clip truncation operation to mitigate the imbalance caused by differences in area size. The expression is shown as below:

(16) $$ClipHaus\left(X,Y\right)=\min\left(\max_{x\in X}\left(\min_{y\in Y}{\left|x-y\right|}_{2}\right),\epsilon \right)$$

Considering that the Hausdorff distance essentially measures the Euclidean distance between pixels, it is crucial to design the constant ϵ related to the Euclidean distance between pixels to maintain the consistency of the distance metric. Additionally, since images of different sizes may result in varying ranges of target boundaries, we need to ensure that the value of ϵ can be adjusted with changes in the input image size to fully accommodate the characteristics of different sized images. Thus, ϵ should be designed as a variable proportional to the image size. Moreover, given that medical image segmentation tasks typically involve several different types of targets, and an increase in the number of categories $N_{C}$ may lead to more complex boundary cases, the value of ϵ should also be inversely proportional to the number of categories $N_{C}$. In this way, we adjust the value of ϵ according to the size of the input image and the number of categories, allowing for the effective evaluation and optimization of segmentation results in various scenarios.

Based on the above considerations, we define ϵ as follows:

(17) $$\epsilon =\frac{\sqrt{H^{2}+W^{2}}}{N_{C}}$$

where *H*, *W*, and $N_{C}$ denote the height, width, and total number of all categories of the input image, respectively.

In summary, our total loss is defined as follows:

(18) $$L_{total}=\frac{1}{\omega_{1}+\omega_{2}+\omega_{3}}\left(\omega_{1}L_{CE}+\omega_{2}L_{Dice}+\omega_{3}L_{ClipHaus}\right)$$

(19) $$L_{ClipHaus}=\max\left(ClipHaus\left(P,G\right),ClipHaus\left(G,P\right)\right)$$

where $\omega_{1}$, $\omega_{2}$, and $\omega_{3}$ serve as hyperparameter weights and are set to 10, 10, and 1, respectively.

## 4. Experiments

### 4.1. Experimental Setup

The experiments were conducted on an Intel Core i9-12900H CPU and NVIDIA GeForce RTX3060 with 6 GB of video memory. The compilation environment for the experiments was pytorch1.13 as well as python3.9, and CUDA11.6 was used for GPU acceleration. A Stochastic Gradient Descent (SGD) optimizer was used in the experiments with momentum parameter set to 0.9, the weight decay rate set to $1\times 10^{-4}$, and the batchsize taken as 2. Learning rate decay was applied during training, following the formula:

(20) $$lr=initial\text{\_}lr{\left(1-\frac{epoch}{max\text{\_}epoch}\right)}^{\alpha }$$

where initial_lr represents the initial learning rate set to 0.01, lr denotes the current learning rate, epoch indicates the current number of training rounds, max_epoch denotes the maximum number of training rounds set to 200, and the decay coefficient α is 0.9. We tried multiple combinations of hyperparameters, including the optimizer, initial learning rate, weight decay, and the depth and width of the network and selected the optimal ones. The model we trained represents the best performance we could achieve.

### 4.2. Dataset

**Figure 5:**
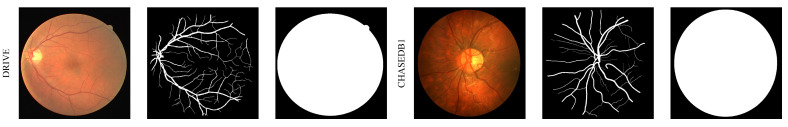
Samples of DRIVE and CHASEDB1 dataset.

Our proposed method was trained and tested on the DRIVE [30] and CHASEDB1 [31] datasets. Example images of both datasets are shown in Figure 5, including the original images, ground truth segmentation masks, and field-of-view (FOV) masks from left to right. The DRIVE dataset comprises 40 real fundus color images, each with the size of 565 × 584 pixels, containing 33 normal images and 7 with pathology. We used 20 images for training and another 20 for testing. Each image includes a blood vessel mask manually labeled by two medical experts, with the first expert’s annotation utilized uniformly as ground truth masks for the segmentation algorithm. The CHASEDB1 dataset consists of 28 high-resolution fundus color images, each sized at 999 × 960 pixels. The dataset encompasses images from 14 healthy individuals and 14 patients with diabetic retinopathy. Similar to DRIVE, each image has two versions with independent manual annotations from different physicians. For our experiments, we divided the dataset by using the first 20 images for training and the last 8 for testing, all based on the first annotation. The division of the training set and testing set follows CoVi-Net [25].

### 4.3. Image Preprocessing

**Figure 6:**
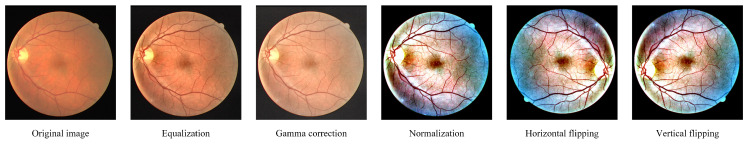
Examples of image preprocessing methods.

In this paper, we employed several image preprocessing methods to further enhance the clarity of the retinal vascular structure. Firstly, to ensure consistency in the input image size of network and considering computational resource limitations, we resized the training and testing set images to 448 × 448. Given the wide range of luminance dynamics and local contrast variations inherent in retinal images, particularly around vessel bifurcations and seams, we employed Contrast Limited Adaptive Histogram Equalization (CLAHE) [32] and Gamma correction to enhance the overall brightness and contrast, thereby making the vascular structures clearer. Subsequently, we normalized the images to adjust the pixel values within the range of −1 to 1. Since the retinal blood vessel segmentation dataset is relatively small, to further reduce overfitting and bolster model robustness, we applied random horizontal flipping and random vertical flipping to the training images. The testing dataset was only preprocessed and not flipped. Figure 6 illustrates an example of image preprocessing methods.

### 4.4. Evaluation Metrics

To evaluate the segmentation effect of retinal blood vessel images, we calculated the confusion matrix and recorded the four specific metrics: true positive (TP), true negative (TN), false positive (FP), and false negative (FN). Based on these metrics, we calculated the Accuracy (ACC), Sensitivity (SE), Specificity (SP), and F1 score (F1) using the following formulas:

(21) $$ACC=\frac{TN+TP}{TN+TP+FN+FP}$$

(22) $$SE=\frac{TP}{TP+FN}$$

(23) $$SP=\frac{TN}{TN+FP}$$

(24) $$F1=\frac{2TP}{2TP+FN+FP}$$

## 5. Results

### 5.1. Ablation Experiments

To further assess the contributions of individual modules in TD Swin-UNet, we conducted ablation experiments using the DRIVE dataset. Swin-UNet served as the baseline, and four evaluation metrics including ACC, SE, SP, and F1 were recorded during the experiments. Table 1 presents the results of seven sets of ablation experiments: baseline + CTCM, baseline + PTHM, baseline + Hausdorff_loss, baseline + CTCM + PTHM, baseline + PTHM+Hausdorff_loss, baseline + CTCM + Hausdorff_loss, and overall improvement.

1. Efficacy of the Cross-level Texture Complementary Module

To further validate the efficacy of CTCM, we compared baseline + CTCM with the baseline alone. In comparison to the baseline, CTCM demonstrated improvements of 0.92%, 4.19%, 0.45%, and 3.77% in ACC, SE, SP, and F1, respectively. As the size of the feature maps decreased during downsampling, the baseline tended to lose shallow details, which contained richer semantic features of small regions and subtle blood vessel branching information. This led to challenges in effectively segmenting blood vessel edges and fine branches, resulting in relatively lower metrics. Incorporating CTCM enhanced the segmentation accuracy in both the blood vessel region (SE) and the background region (SP), leading to more precise recognition of blood vessels.

2. Efficacy of the Pixel-wise Texture Highlighting Module

To further confirm the effectiveness of PTHM, we integrated PTHM into the baseline. In comparison to the baseline alone, PTHM yielded improvements of 1.11%, 3.89%, 0.71%, and 4.32% in ACC, SE, SP, and F1, respectively. The incorporation of the improved PT Swin Block in the upsampling process enhanced the model’s ability to perceive the vessel boundary and contour information. The concurrent enhancement of SE and SP indicated that the enhancement module effectively learned semantic information on both sides of the boundary, leading to a significant improvement in the connectivity and wholeness of vessel segmentation.

3. Efficacy of Improved Hausdorff Loss

To address the insensitivity of the original loss function to specific boundary shapes and distributions, we further introduced improved Hausdorff loss into the baseline. The integration of Hausdorff loss resulted in improvements of 0.79%, 3.65%, 0.38%, and 3.24% in ACC, SE, SP, and F1, respectively. Hausdorff_loss more accurately reflected the distance between the predicted boundary and the real boundary, and strengthened the model’s ability to learn the specific boundary shape and distribution characteristics of the blood vessels. Consequently, the segmentation accuracy of the blood vessel region was significantly improved, leading to an overall enhancement in blood vessel segmentation effectiveness.

4. Efficacy of Overall Improvements

By integrating the above improvement modules, the final configuration of baseline + CTCM + PTHM + Hausdorff_loss achieved 96.64%, 84.49%, 98.37%, and 86.53% on ACC, SE, SP, and F1, respectively. Compared to baseline + PTHM + Hausdorff_loss, baseline + CTCM + PTHM + Hausdorff_loss exhibited a slight decrease of 0.74% in SE, but witnessed increases in SP and F1 from 97.67% and 84.90%, to 98.37% and 86.53%, resulting in an overall enhancement in vessel segmentation performance. In addition, compared to baseline + CTCM + Hausdorff_loss, baseline + CTCM + PTHM + Hausdorff_loss resulted in a marginal decrease of 0.2% in SP, yet SE and F1 improved by 2.04% and 0.6%, leading to an improved overall blood vessel segmentation performance. Despite a slight reduction in the background region segmentation accuracy, the accuracy of blood vessel segmentation and the overall performance was greatly improved. In conclusion, TD Swin-UNet effectively achieved accurate segmentation of complex structured blood vessel images, and finally exhibited high segmentation accuracy.

### 5.2. Visualization Results

**Figure 7:**
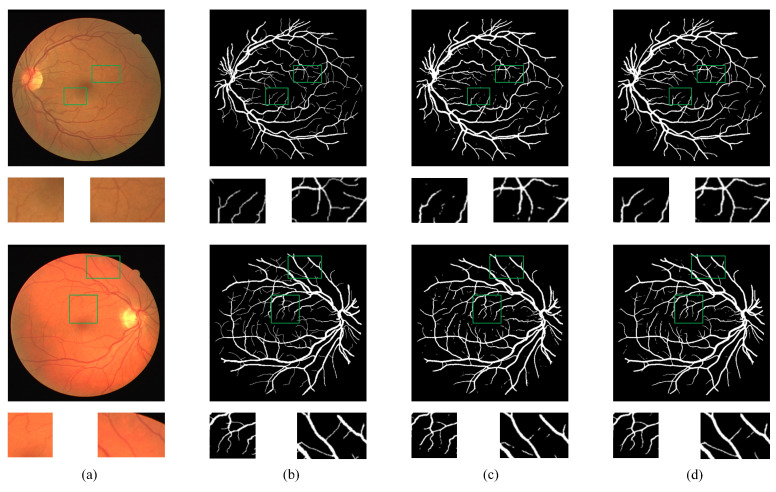
Samples of visualization results on DRIVE. (**a**) Original image; (**b**) ground truth; (**c**) baseline; (**d**) proposed.

**Figure 8:**
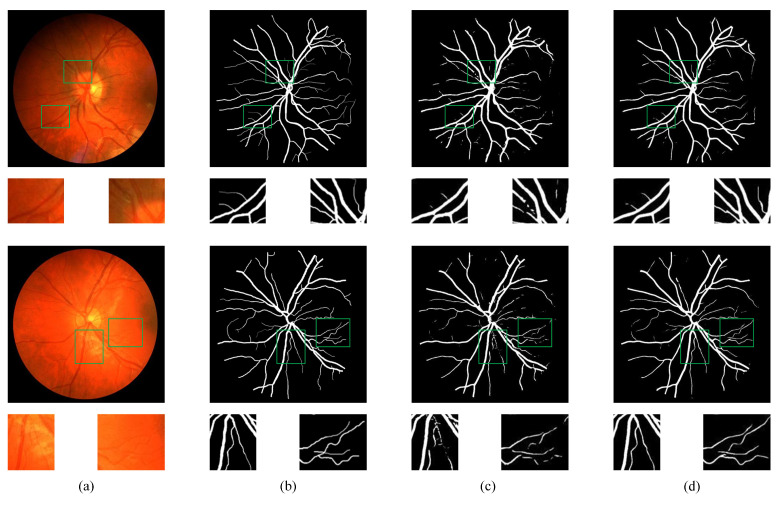
Samples of visualization results on CHASEDB1. (**a**) Original image; (**b**) ground truth; (**c**) baseline; (**d**) proposed.

Figure 7 and Figure 8 depict the visualized segmentation results of the baseline and our proposed method on the DRIVE and CHASEDB1 datasets, respectively. These figures showcase the overall segmentation results of retinal vessel structures alongside localized zoomed-in images for a detailed examination of vessel segmentation. Both approaches effectively capture the main branches of thicker blood vessels in the retinal images. However, the retinal blood vessel images also have complex and intertwined fine branch structures. Due to the lack of a unique feature fusion mechanism and edge enhancement module, the baseline method had challenges in the localization and segmentation of fine blood vessels, leading to imprecise vessel boundary delineation. In comparison, our proposed model exhibited enhanced boundary detection capabilities, enabling more accurate vessel boundary delineation. Notably, as highlighted by the green box, the baseline method fared poorly in segmenting fine blood vessels characterized by discontinuous vessel structures. In contrast, the proposed method effectively captured the detailed texture information at vessel boundaries, resulting in accurate and continuous segmentation outcomes. The visualization results demonstrate the superiority of the model in localizing and segmenting vessel boundaries, particularly in accurately segmenting fine blood vessels with complex structures.

### 5.3. Comparisons with Existing Methods

To further validate the superiority of TD Swin-UNet, we compared it with 13 retinal vessel segmentation methods proposed over the past ten years on the DRIVE and CHASEDB1 datasets. These methods include SegNet [8], UNet [9], Att-Unet [33], UNet++ [11], CE-Net [34], AA-UNet [20], Efficient BFCN [35], PSP-UNet [36], AMF-NET [37], IterNet++ [38], TiM-Net [39], CAS-UNet [40], and LMSA-Net [41]. We conducted comparative experiments on the first five methods, employing identical training strategies and environments across all experiments. For the latter eight methods, due to the lack of open-source code, all data are cited directly from the original texts. Table 2 and Table 3 present the comparison results on the DRIVE and CHASEDB1 datasets, with the experimental metrics including ACC, SE, SP, and F1, where “-” indicates that the experimental data for the item were not available in the original literature.

On the DRIVE dataset, TD Swin-UNet achieved the highest SE, Specificity SP, and F1, reaching 0.8479, 0.9837, and 0.8653, respectively. Despite LMSA-Net [41] having a slightly higher ACC than our model (by 0.22%), TD Swin-UNet outperformed it with SE, SP, and F1 values that were higher by 1.71%, 0.16%, and 4.39%, respectively. The increase in SE and SP signifies the enhanced accuracy in retinal vessel identification. Although the improvement in SP was relatively small, our model had a significantly higher accuracy for SE in vessel region segmentation. The introduction of CTCM and PTHM restored lost boundary information during downsampling and effectively improved the model’s ability to perceive the boundary and contour information of the blood vessels, leading to more accurate segmentation and increased vascular connectivity and wholeness.

On the CHASEDB1 dataset, TD Swin-UNet achieved the highest ACC, SE, and F1, which were improved by 0.05%, 0.9%, and 1.25% respectively, compared with the maximum values of the other models. Although the SP of TD Swin-UNet (0.9867) was slightly lower than that of AMF-Net [37] (0.9881), TiM-Net [39] (0.9880), and CAS-UNet [40] (0.9896), TD Swin-UNet achieved a significant improvement in the SE of the vessel region segmentation accuracy due to the attention and enhancement of the detailed texture features near the vessel boundary, resulting in the highest ACC (0.9756) and F1 (0.8515). Considering the substantial improvement in SE, the slight deficiency in SP became negligible. TD Swin-UNet demonstrated more accurate segmentation of blood vessels and background regions compared with the other methods, making it more suitable for clinical applications in medical imaging and showing promising prospects in various fields.

**Figure 9:**
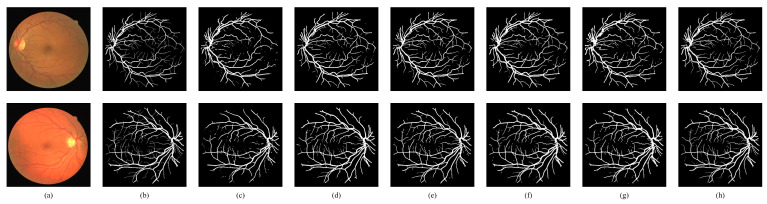
Visual comparisons with other methods on DRIVE. (**a**) Original image; (**b**) ground truth; (**c**) SegNet; (**d**) UNet; (**e**) Att-UNet; (**f**) UNet++; (**g**) CE-Net; (**h**) proposed.

**Figure 10:**
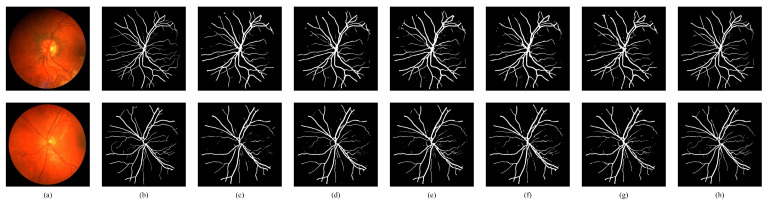
Visual comparisons with other methods on CHASEDB1. (**a**) Original image; (**b**) ground truth; (**c**) SegNet; (**d**) UNet; (**e**) Att-UNet; (**f**) UNet++; (**g**) CE-Net; (**h**) proposed.

We also compared the visualization results of the proposed model with five other methods: SegNet [8], UNet [9], Att-Unet [33], UNet++ [11], and CE-Net [34]. Figure 9 and Figure 10 depict the visual comparisons on the DRIVE and CHASEDB1 datasets. While SegNet exhibited poor segmentation performance with notable background noise, UNet and Att-UNet achieved accurate segmentation of major arteries and veins, but struggled with finer blood vessel branches. UNet++ introduced a dense connection mechanism and depth supervision, which had a better segmentation effect on the local blood vessels. However, it fell short in capturing global information, and holistic blood vessel segmentation needs to be improved. CE-Net introduces a contextual feature extraction module consisting of DAC and RMP on the basis of UNet to fuse multi-scale contextual information, but it struggled to effectively capture the semantic information of the vessel structure, resulting in discontinuous vessel segmentation. In contrast, the proposed TD Swin-UNet effectively captured the long-range dependencies of blood vessels, leading to more connected vessel segmentation results. In addition, due to the introduction of CTCM and PTHM, the proposed model was able to accurately segment the details at the vessel boundary, yielding superior segmentation outcomes.

## 6. Discussions

The proposed model addresses the challenge of vascular detail loss during downsampling in the baseline model, yielding notable advancements in the segmentation of retinal vascular detail branches and boundaries. However, several limitations still persist. Primarily, concerning model complexity, the proposed method requires an average inference time of 2.87 s for processing a single image from the DRIVE dataset and 2.54 s for the CHASEDB1 dataset. This poses a challenge in achieving the real-time segmentation of retinal vascular images, potentially impacting the efficiency of disease diagnosis and treatment decision-making by clinicians in practical scenarios. Additionally, due to constraints in imaging technology during data acquisition, there exists a significant resolution gap between retinal vessel images in the DRIVE and STARE datasets and that of contemporary fundus vascular imaging systems. The increase in the input image resolution would substantially escalate the demands on model memory and computational resources, thereby imposing a considerable computational burden and potentially compromising the responsiveness and stability of the segmentation system.

## 7. Conclusions

For the accurate segmentation of retinal vessel images, we propose a texture-driven retinal vessel segmentation method by improving boundary-wise perception. Built upon Swin-UNet, our method integrates three pivotal improvement modules: the CTCM, PTHM, and improved Hausdorff loss. In the encoder stage, the CTCM consolidates feature maps across different scales to reinstate lost detailed features during downsampling and prioritize key features within the image. In the decoder stage, the PTHM is combined with the Swin Block to form the PT Swin Block, which helps the model to perceive the detailed texture information of the boundary region and further refines the boundary localization accuracy. The incorporation of improved Hausdorff loss addresses the insensitivity of the original model to specific boundary shapes and distributions, enabling the model to capture subtle boundary differences and improve segmentation accuracy. Experimental results show that TD Swin-UNet exhibits a superior segmentation accuracy and boundary localization capability compared to other retinal vessel segmentation algorithms. However, real-time performance is a major drawback of TD Swin-UNet. In the future, we will optimize the model architecture to achieve a more lightweight network, reducing both the model parameter count and computational costs. Additionally, our model was only applied in the domain of retinal vessel segmentation and was not tested on other types of medical images. Moving forward, we aim to apply this model to various medical image datasets and optimize it into a unified, highly generalizable medical segmentation network, better facilitating the advancement of automated medical analysis.

## Figures and Tables

**Table 1:** Ablation studies of the proposed method on DRIVE.

	CTCM	PTHM	Hausdorff	ACC	SE	SP	F1
baseline				0.9466	0.7817	0.9706	0.7883
	✓			0.9558	0.8236	0.9751	0.8260
Combination		✓		0.9577	0.8206	0.9777	0.8315
of each			✓	0.9545	0.8182	0.9744	0.8207
improvement	✓	✓		0.9641	0.8443	0.9816	0.8569
		✓	✓	0.9613	**0.8553**	0.9767	0.8490
	✓		✓	0.9655	0.8274	**0.9857**	0.8593
Ours	✓	✓	✓	**0.9664**	0.8479	0.9837	**0.8653**

Bolded data indicate the optimal values for the current indicators.

**Table 2:** Comparison of the proposed method with other methods on DRIVE.

Method	Year	ACC	SE	SP	F1
SegNet [8]	2015	0.9349	0.6850	0.9714	0.7284
UNet [9]	2015	0.9531	0.7923	0.9765	0.8113
Att-UNet [33]	2018	0.9512	0.7829	0.9757	0.8032
UNet++ [11]	2019	0.9552	0.8046	0.9771	0.8205
CE-Net [34]	2019	0.9408	0.7931	0.9623	0.7733
AA-UNet [20]	2020	0.9558	0.7941	0.9798	-
Efficient BFCN [35]	2020	0.9627	0.8124	0.9822	0.8294
PSP-UNet [36]	2021	0.9556	0.7814	0.9810	0.8176
AMF-NET [37]	2021	0.9581	0.8139	0.9826	-
IterNet++ [38]	2022	0.9569	0.8399	0.9742	-
TiM-Net [39]	2022	0.9638	0.7805	0.9816	-
CAS-UNet [40]	2023	0.9586	0.8375	0.9828	0.8207
LMSA-Net [41]	2023	**0.9686**	0.8308	0.9821	0.8214
ours	2024	0.9664	**0.8479**	**0.9837**	**0.8653**

Bolded data indicate the optimal values for the current indicators.

**Table 3:** Comparison of the proposed method with other methods on CHASEDB1.

Method	Year	ACC	SE	SP	F1
SegNet [8]	2015	0.9560	0.7521	0.9763	0.7559
UNet [9]	2015	0.9591	0.8064	0.9744	0.7822
Att-UNet [33]	2018	0.9581	0.7660	0.9772	0.7679
UNet++ [11]	2018	0.9603	0.8123	0.9750	0.7873
CE-Net [34]	2019	0.9559	0.8131	0.9701	0.7696
AA-UNet [20]	2020	0.9608	0.8176	0.9704	-
Efficient BFCN [35]	2020	0.9688	0.8323	0.9851	0.8102
PSP-UNet [36]	2021	0.9590	0.8195	0.9727	0.7813
AMF-NET [37]	2021	0.9729	0.8344	0.9881	-
IterNet++ [38]	2022	0.9659	0.8247	0.9820	-
TiM-Net [39]	2022	0.9711	0.7867	0.9880	-
CAS-UNet [40]	2023	0.9668	0.8321	**0.9896**	0.8390
LMSA-Net [41]	2023	0.9751	0.8428	0.9840	0.8097
ours	2024	**0.9756**	**0.8518**	0.9867	**0.8515**

Bolded data indicate the optimal values for the current indicators.

## Data Availability

In our experiment, we utilized two publicly available datasets, DRIVE and CHASEDB1. These datasets can be obtained from the following links: https://drive.grand-challenge.org/DRIVE/ (accessed on 15 December 2023), https://blogs.kingston.ac.uk/retinal/chasedb1/ (accessed on 15 December 2023).

## References

[1] Tan Y., Yang K.F., Zhao S.X., Li Y.J. (2022). Retinal Vessel Segmentation with Skeletal Prior and Contrastive Loss. IEEE Trans. Med. Imaging.

[2] Chen C., Chuah J.H., Ali R., Wang Y. (2021). Retinal Vessel Segmentation Using Deep Learning: A Review. IEEE Access.

[3] Jiang X., Mojon D. (2003). Adaptive local thresholding by verification-based multithreshold probing with application to vessel detection in retinal images. IEEE Trans. Pattern Anal. Mach. Intell..

[4] Saroj S.K., Kumar R., Singh N.P. (2020). Fréchet PDF based Matched Filter Ap proach for Retinal Blood Vessels Segmentation. Comput. Methods Programs Biomed..

[5] He K., Zhang X., Ren S., Sun J. Deep Residual Learning for Image Recognition. Proceedings of the IEEE Conference on Computer Vision and Pattern Recognition.

[6] Redmon J., Divvala S., Girshick R., Farhadi A. You Only Look Once: Unified, Real-Time Object Detection. Proceedings of the IEEE Conference on Computer Vision and Pattern Recognition.

[7] Shelhamer E., Long J., Darrell T. (2017). Fully Convolutional Networks for Semantic Segmentation. IEEE Trans. Pattern Anal. Mach. Intell..

[8] Badrinarayanan V., Kendall A., Cipolla R. (2017). SegNet: A Deep Convolutional Encoder-Decoder Architecture for Image Segmentation. IEEE Trans. Pattern Anal. Mach. Intell..

[9] Ronneberger O., Fischer P., Brox T. (2015). U-net: Convolutional networks for biomedical image segmentation. Proceedings of the International Conference on Medical Image Computing and Computer-Assisted Intervention.

[10] Zhao H., Shi J., Qi X., Wang X., Jia J. Pyramid Scene Parsing Network. Proceedings of the IEEE Conference on Computer Vision and Pattern Recognition.

[11] Zhou Z., Siddiquee M.M.R., Tajbakhsh N., Liang J. (2018). Unet++: A nested u-net architecture for medical image segmentation. Proceedings of the Deep Learning in Medical Image Analysis and Multimodal Learning for Clinical Decision Support.

[12] Sathananthavathi V., Indumathi G. (2021). Encoder Enhanced Atrous (EEA) Unet architecture for Retinal Blood vessel segmentation. Cognit. Syst. Res..

[13] Li K., Qi X., Luo Y., Yao Z., Zhou X., Sun M. (2021). Accurate Retinal Vessel Segmentation in Color Fundus Images via Fully Attention-Based Networks. IEEE J. Biomed. Health Inform..

[14] Han J., Wang Y., Gong H. (2022). Fundus Retinal Vessels Image Segmentation Method Based on Improved U-Net. IRBM.

[15] Vaswani A., Shazeer N., Parmar N., Uszkoreit J., Jones L., Gomez A.N., Kaiser L., Polosukhin I. (2017). Attention Is All You Need. arXiv.

[16] Wang X., Girshick R., Gupta A., He K. Non-local Neural Networks. Proceedings of the 2018 IEEE/CVF Conference on Computer Vision and Pattern Recognition.

[17] Dosovitskiy A., Beyer L., Kolesnikov A., Weissenborn D., Zhai X., Unterthiner T., Dehghani M., Minderer M., Heigold G., Gelly S. (2020). An Image is Worth 16 × 16 Words: Transformers for Image Recognition at Scale. arXiv.

[18] Liu Z., Lin Y., Cao Y., Hu H., Wei Y., Zhang Z., Lin S., Guo B. Swin transformer: Hierarchical vision transformer using shifted windows. Proceedings of the IEEE/CVF International Conference on Computer Vision 2021.

[19] Cao H., Wang Y., Chen J., Jiang D., Zhang X., Tian Q., Wang M. (2021). Swin-unet: Unet-like pure transformer for medical image segmentation. arXiv.

[20] Lv Y., Ma H., Li J., Liu S. (2020). Attention Guided U-Net with Atrous Convolution for Accurate Retinal Vessels Segmentation. IEEE Access.

[21] Yang X., Liu L., Li T. (2022). MR-UNet: An UNet model using multi-scale and residual convolutions for retinal vessel segmentation. Int. J. Imaging. Syst. Technol..

[22] Radha K., Karuna Y. (2023). Modified Depthwise Parallel Attention UNet for Retinal Vessel Segmentation. IEEE Access.

[23] Cao J., Chen J., Gu Y., Liu J. (2023). MFA-UNet: A vessel segmentation method based on multi-scale feature fusion and attention module. Front. Neurosci..

[24] Jiang Y., Liang J.Q., Cheng T., Lin X., Zhang Y., Dong J. (2022). MTPA Unet: Multi-Scale Transformer-Position Attention Retinal Vessel Segmentation Network Joint Transformer and CNN. Sensors.

[25] Jiang M., Zhu Y., Zhang X. (2024). CoVi-Net: A hybrid convolutional and vision transformer neural network for retinal vessel segmentation. Comput. Biol. Med..

[26] Jia W., Ma S., Geng P., Sun Y. (2023). DT-Net: Joint Dual-Input Transformer and CNN for Retinal Vessel Segmentation. CMC-Comput. Mater. Contin..

[27] Tan X., Chen X., Meng Q., Shi F., Xiang D., Chen Z., Pan L., Zhu W. (2023). OCT2Former: A retinal OCT-angiography vessel segmentation transformer. Comput. Methods Programs Biomed..

[28] Wang T., Dai Q. (2023). SURVS: A Swin-Unet and game theory-based unsupervised segmentation method for retinal vessel. Comput. Biol. Med..

[29] Lin J., Huang X., Zhou H., Wang Y., Zhang Q. (2023). Stimulus-guided adaptive transformer network for retinal blood vessel segmentation in fundus images. Med. Image Anal..

[30] Staal J., Abramoff M., Niemeijer M., Viergever M., van Ginneken B. (2004). Ridge-based vessel segmentation in color images of the retina. IEEE Trans. Med. Imaging.

[31] Owen C.G., Rudnicka A.R., Mullen R., Barman S.A., Monekosso D.N., Whincup P.H., Ng J., Paterson C. (2009). Measuring retinal vessel tortuosity in 10-year-old children: Validation of the Computer-Assisted Image Analysis of the Retina (CAIAR) program. Investig. Ophthalmol. Vis. Sci..

[32] Pizer S.M., Amburn E.P., Austin J.D., Cromartie R., Geselowitz A., Greer T., ter Haar Romeny B., Zimmerman J.B., Zuiderveld K. (1987). Adaptive histogram equalization and its variations. Comput. Vis. Graph. Image Process..

[33] Oktay O., Schlemper J., Folgoc L.L., Lee M.J., Heinrich M.P., Mis-awa K., Mori K., McDonagh S.G., Hammerla N.Y., Kainz B. (2018). Attention U-Net: Learning Where to Look for the Pancreas. arXiv.

[34] Gu Z., Cheng J., Fu H., Zhou K., Hao H., Zhao Y., Zhang T., Gao S., Liu J. (2019). CE-Net: Context Encoder Network for 2D Medical Image Segmentation. IEEE Trans. Med. Imaging.

[35] Yun J., Wang F., Gao J., Liu W. (2022). Efficient BFCN for Automatic Retinal Vessel Segmentation. J. Ophthalmol..

[36] Du X.F., Wang J.S., Sun W.Z. (2021). UNet retinal blood vessel segmentation algorithm based on improved pyramid pooling method and attention mechanism. Phys. Med. Biol..

[37] Yang Q., Ma B., Cui H., Ma J. AMF-NET: Attention-Aware Multi-Scale Fusion Network for Retinal Vessel Segmentation. Proceedings of the 2021 43rd Annual International Conference of the IEEE Engineering in Medicine & Biology Society (EMBC).

[38] Zhu M., Zeng K., Lin G., Gong Y., Hao T., Wattanachote K., Luo X. (2022). IterNet++: An improved model for retinal image segmentation by curvelet enhancing, guided filtering, offline hard-sample mining, and test-time augmenting. IET Image Process.

[39] Zhang H., Zhong X., Li Z., Chen Y., Zhu Z., Lv J., Li C., Zhou Y., Li G. (2022). TiM-Net: Transformer in M-Net for Retinal Vessel Segmentation. J. Healthc. Eng..

[40] You Z., Yu H., Xiao Z., Peng T., Wei Y. (2023). CAS-UNet: A Retinal Segmentation Method Based on Attention. Electronics.

[41] Chen J., Wan J., Fang Z., Wei L. (2023). LMSA-Net: A lightweight multi-scale aware network for retinal vessel segmentation. Int. J. Imaging Syst. Technol..

